# Cerebral Autoregulation Is Minimally Influenced by the Superior Cervical Ganglion in Two- Week-Old Lambs, and Absent in Preterm Lambs Immediately Following Delivery

**DOI:** 10.1371/journal.pone.0082326

**Published:** 2013-12-09

**Authors:** Adam J. Czynski, Michael H. Terry, Douglas D. Deming, Gordon G. Power, John N. Buchholz, Arlin B. Blood

**Affiliations:** 1 Department of Pediatrics, Division of Neonatology, Loma Linda University School of Medicine, Loma Linda, California, United States of America; 2 Center for Perinatal Biology, Loma Linda University School of Medicine, Loma Linda, California, United States of America; 3 Department of Physiology and Pharmacology, Loma Linda University School of Medicine, Loma Linda, California, United States of America; 4 Department of Respiratory Care, Pulmonary Physiology Laboratories, Loma Linda University Medical Center, Loma Linda, California, United States of America; Scuola Superiore Sant’Anna Institute of Life Sciences, Italy

## Abstract

Cerebral vessels in the premature newborn brain are well supplied with adrenergic nerves, stemming from the superior cervical ganglia (SCG), but their role in regulation of blood flow remains uncertain. To test this function twelve premature or two-week-old lambs were instrumented with laser Doppler flow probes in the parietal cortices to measure changes in blood flow during changes in systemic blood pressure and electrical stimulation of the SCG. In lambs delivered prematurely at ∼129 days gestation cerebral perfusion and driving pressure demonstrated a direct linear relationship throughout the physiologic range, indicating lack of autoregulation. In contrast, in lambs two-weeks of age, surgical removal of one SCG resulted in ipsilateral loss of autoregulation during pronounced hypertension. Electrical stimulation of one SCG elicited unilateral increases in cerebral resistance to blood flow in both pre-term and two-week-old lambs, indicating functioning neural pathways in the instrumented, anesthetized lambs. We conclude cerebral autoregulation is non-functional in preterm lambs following cesarean delivery. Adrenergic control of cerebral vascular resistance becomes effective in newborn lambs within two-weeks after birth but SCG-dependent autoregulation is essential only during pronounced hypertension, well above the normal range of blood pressure.

## Introduction

Under physiological conditions, cerebral vessels compensate for increases and decreases in perfusion pressure by changing caliper, resulting in a relatively constant blood flow across a range of pressures. This phenomenon is called cerebral autoregulation and has been demonstrated in adults, newborns, and even in fetal sheep *in utero* as young as 92 days of gestation (term 147) [1]. At least three mechanisms have been proposed to contribute to autoregulation of cerebral blood flow (CBF) [2]: 1) local tissue metabolic feedback such as tissue carbon dioxide and oxygen tensions, and adenosine; 2) intrinsic myogenic responses of resistance arterioles to changes in transmural pressure; and 3) neurogenic input from the central innervation of the cerebral arteries. While the first two mechanisms are widely accepted, the overall importance of the neurogenic control of autoregulation has been a topic of uncertainty for many decades [3].

The extra-parenchymal arteries supplied by the Circle of Willis, including the pial arterioles, are richly innervated by sympathetic nerve fibers [4]–[6] that derive from the SCG [7]. Studies to determine whether these nerves facilitate autoregulation have yielded conflicting results. Removal of the SCG increases baseline CBF in newborn piglets [8] and increases CBF in adult primates, dogs, and cats during acute hypertension [7], [9]. In addition, electrical stimulation of the SCG reduces CBF and pial arteriolar diameter in a number of animal species [9]–[17]. In contrast, others have observed no effect on cerebral blood flow following acute sympathetic denervation in cats [18] chronic denervation in primates [19] and cats [20], or during sympathetic activation in humans [21]. Studies in preterm fetal lambs indicate that stimulation of the SCG causes pial artery vasoconstriction as early as 121 days gestation [17], a result in agreement with reported studies in newborn lambs [15] and piglets [16]. Recent work also suggests that sympathetic nervous system activity through the SCG is involved in regulation of CBF during blood pressure fluctuations during periods of REM sleep in newborn lambs [22], [23].

Preterm infants are particularly susceptible to dysregulation of CBF and suffer from intracerebral hemorrhage [24] and periventricular leukomalacia [25], [26]. Therefore, we evaluated the influence of the sympathetic system on CBF in premature lambs following cesarean delivery. We test the null hypothesis that the SCG does not influence CBF in response to 1) direct electrical stimulation of the SCG or 2) acute changes in mean arterial pressure. This hypothesis was tested in both premature and two-week-old newborn lambs.

## Materials and Methods

### Surgical Preparation

Animal protocols were pre-approved by the Institutional Animal Care and Use Committee of Loma Linda University. All sheep were mixed Western breed. Experiments were conducted on either preterm lambs (*n* = 6) that were delivered by C-section on gestational day 127 to 132 (term 147) or on two-week-old lambs (*n* = 6) following natural delivery. Anesthesia was induced in the ewes (for the preterm lamb group) and two-week-old lambs with an intra-jugular injection of thiopental sodium (10 mg/kg), followed by intubation and mechanical ventilation with 2% isoflurane throughout the surgical instrumentation. The umbilical cord of the preterm lamb was kept intact during the surgery with only the head and thorax exteriorized through a midline incision in the maternal abdomen.

Both preterm and two-week-old lambs were instrumented with bilateral laser Doppler flow probes (LDF, Oxford Optronix Ltd, Oxford, UK), inserted through 1.5 mm burr holes drilled 5 mm lateral to the sagittal suture and 15 mm posterior to the coronal suture [27]. The probes were inserted to a depth of 10 mm below the outer surface of the skull. This method of CBF measurement has been validated against microspheres previously in this animal model [27], [28] and provides a continuous measurement of relative changes in blood perfusion of a ∼2 mm^3^ volume of tissue surrounding the tip of the probe. Laser Doppler flow probes were fixed in place, and then four hours were allowed to elapse for blood flow to reach a stable baseline prior to the start of the study protocol. All lambs were also instrumented with snare occluders placed around the brachiocephalic trunk and descending aorta. Access to these vessels was obtained through a left thoracotomy and removal of the 5^th^ rib. Catheters were inserted into the brachial artery and vein for measurement of arterial blood pressure and gases and infusion of medications, respectively. Following surgical instrumentation, preterm lambs were intubated via tracheostomy, delivered, the cord ligated, and ventilation begun as described below. After completion of the surgical procedures for both preterm and two week old lambs, isoflurane was discontinued and anesthesia was converted to intravenous ketamine (1 mg kg^−1^ hr^−1^) and pancuronium (0.1 mg kg^−1^ hr^−1^).

Both preterm and two-week-old lambs were mechanically ventilated (VIP Bird, CareFusion Inc., Yorba Linda, CA) with initial settings at a peak inspiratory pressure of 25 cm H_2_O, positive end-expiratory pressure of 5 cm H_2_O, inspiratory:expiratory ratio time of 1:3, and respiratory rate of 60 bpm, with subsequent adjustments made to target an arterial PO_2_ of ∼200 Torr and PCO_2_ of 35 to 45 Torr. The target arterial PO_2_ was set higher than the physiologic range to overcome ventilation-perfusion mismatch in the lambs and thereby to ensure cerebral autoregulation was not influenced by hypoxia. Trometamol; tris-hydroxymethyl aminomethane (THAM, 0.3 M, 12 ml/kg pH 8.6) was administered to treat metabolic acidosis as needed. Rectal temperature was maintained at 39±1 °C by use of a radiant warmer (4400 Infant Warmer, Ohmeda, Madison, WI).

Lambs were placed in the supine position and both SCG were exposed via an incision from the angle of the mandible to the transverse process of the second cervical vertebrae. One SCG was randomly chosen for insertion of two platinum wire stimulating electrodes connected to a Grass S-48 model stimulator (Grass Instruments, Quincy, MA).

### Experimental Protocol

Baseline measurements were made after a four-hour recovery period following placement of the LDF probes. The lambs where then given an intravenous bolus of norepinephrine (5 μg/kg) and angiotensin II (10 μg/kg) to cause hypertension. Systemic hypertension was obtained by pharmacological means because it enabled the study of higher arterial blood pressures than could be obtained by occlusion of the descending aorta, and with the rationale that a favorable comparison with mechanical means would strengthen interpretation of the results. Each bolus was followed by a 5- to 15- minute recovery period until blood pressure returned to baseline. Following pharmacologically induced hypertension lambs were subjected to 100% occlusion of the brachiocephalic trunk for 30 seconds to reduce cerebral perfusion pressure. This was followed by 3 to 5 minutes of recovery to return to a stable baseline. Next, a graded 30-second occlusion was administered to generate a fall in arterial pressure equivalent to 50% of the decrease caused by the 100% occlusion, followed by another recovery period. Finally, a third occlusion was administered to achieve a 25% decrease in arterial pressure compared to the 100% occlusion. The lambs were then subjected to a similar series of 100%, 50%, and 25% occlusions of the descending aorta to generate an increase in pressure perfusing the cerebral circulation. Next, to assess the effects of electrical stimulation of the SCG, electrical impulses were administered directly to the SCG, at an interval of 1 millisecond with a frequency of 8 Hz and intensity of 6 to 8 volts. A total of four stimulations (two at 6 volts and two at 8 volts) were performed on each lamb. Each stimulation lasted for 30 seconds and was followed by a 3- to 5-min recovery period. To confirm that CBF responses were through the SCG itself in three lambs the surrounding tissue was stimulated and found to be without effect.

Lastly, the SCG that had been instrumented with electrodes was surgically removed and occlusions of the brachiocephalic trunk and descending aorta were repeated, as were the infusions of angiotensin II and norepinephrine. After completion of the experiment, the lambs were euthanized with Euthasol® (Western Medical Supply, Arcadia, CA).

### Data Collection and Analysis

Arterial blood pressure, heart rate, and CBF were recorded digitally at 400 Hz using an A/D converter (Powerlab 16, ADInstruments, Grand Junction, CO) and software (Labchart 6.0, ADInstruments). Because LDF provides only relative changes in flow, CBF and cerebral vascular resistance were expressed as a percent of baseline values. Cerebral vascular resistance was calculated as mean arterial blood pressure divided by CBF and also normalized to baseline values. The effect of electrical stimulation was measured by averaging CBF signals into 5-second blocks, beginning 30 seconds prior to the beginning of the electrical stimulation and continuing until 30 seconds after the end of the stimulation.

Cerebral blood flow and arterial blood pressure during the last 15 seconds of each graded occlusion of the brachiocephalic trunk and aorta, and at the peak response to norepinephrine and angiotensin II injection, were normalized to the 30 seconds of data recorded just prior to each intervention. Autoregulation of CBF was then evaluated by comparison of CBF with mean arterial blood pressure in 2.5 mmHg intervals as previously described [28].

### Statistical Analyses

**Figure 1:**
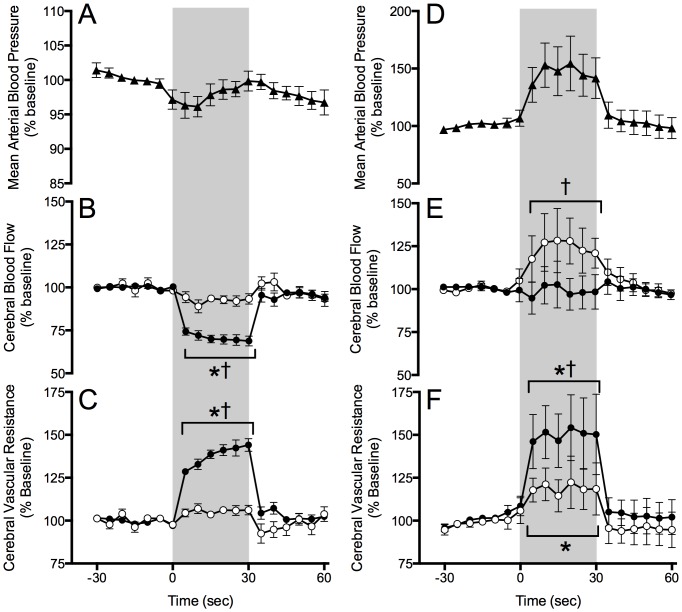
Effect of electrical stimulation of the SCG, shown by the vertical shaded band on mean arterial blood pressure (▴) and ipsilateral (•) and contralateral (○) CBF and cerebral vascular resistance. In preterm lambs (A, B, and C, *n*  =  6), SCG stimulation decreased ipsilateral CBF and increased vascular resistance compared to contralateral values. There were no significant changes in mean arterial blood pressure or contralateral blood flow and vascular resistance over time. In two-week-old lambs (D, E, and F, *n*  =  6), stimulation increased arterial blood pressure and contralateral CBF compared to baseline. Stimulation in two-week-old lambs increased ipsilateral cerebral vascular resistance compared to both baseline and the contralateral cortex. *  =  significant difference from baseline values, p<0.05, †  =  significant difference from contralateral side, p<0.05, data are mean ± SE.

A one-way ANOVA was used to assess the significance of the effects of electrical stimulation of the SCG on mean arterial blood pressure. The effects of stimulation on ipsilateral vs. contralateral (with respect to the side of SCG stimulation) CBF and cerebral vascular resistance time series data (as shown in Figure 1) were compared using a two-way ANOVA. To determine whether comparable changes in blood pressure were administered before and after unilateral SCG removal, pre- and post-SCG removal blood pressure changes were compared by unpaired t-test. Comparison of CBF responses to blood pressure interventions were made for ipsilateral vs. contralateral and pre- vs. post-SCG removal by one-way ANOVA for each blood pressure intervention, with Bonferroni’s post hoc analysis where appropriate. To evaluate the role of the SCG in autoregulation, ipsilateral and contralateral cerebral blood flows were plotted against arterial blood pressure and analyzed using least-squares regression. For each dataset, the goodness of fit to a straight line or third order equation was compared using an extra sum-of-squares F test. The equation with the best fit was then plotted with 95% confidence intervals. All statistical comparisons were performed using Prism 6.0 for Mac (Graphpad Software, La Jolla, CA), and were considered significant at p<0.05. Sensitivity power analysis of the data following completion of the study (G*Power v.3.1.2; University of Dusseldorf, Dusseldorf, Germany) indicated that the study was adequately powered with *n*  =  6 for an α of 0.05 and a β of 0.20. All data are presented as mean ± SE.

## Results

### Animal information

A total of six preterm lambs were studied at an average gestational age of 129±1 days. A total of six two-week-old lambs were studied at an average postnatal age of 13±1 days. Baseline arterial blood gases are shown in Table 1, and did not change significantly over the course of the study in either group (data not shown, one-way ANOVA with repeated measures). The ratio of right vs left SCG randomly chosen for stimulation and subsequent removal was 3/3 for preterm lambs, and 2/4 for two-week-old lambs.

**Table 1:** Baseline* arterial blood gases and hemoglobin.

	Preterm lambs	Two-week-old old lambs
PCO_2_ (Torr)	43±2	41±2
pH	7.36±0.06	7.38±0.04
PO_2_ (Torr)	193±36	204±24
Hemoglobin (g/dL)	11.5±1	9.2±1

* Baseline measurements were performed within five minutes prior to initiation of the initial blood pressure intervention. Mean ± SEM.

### Superior cervical ganglion stimulation

To assess the potential for modulation of CBF by the SCG, 30 second electrical stimulations were applied to one SCG while CBF was monitored in both sides of the brain. In preterm lambs 30 seconds of SCG stimulation decreased blood flow in the ipsilateral cerebral cortex while there was no significant change in flow to the contralateral cortex. Systemic blood pressure remained unchanged (Figure 1). Calculated cerebral vascular resistance rose significantly in the ipsilateral cortex but was not changed in the contralateral cortex. Thus electrical activity stemming from the SCG was able to modulate CBF in these anesthetized and instrumented preterm lambs.

In two-week-old lambs SCG stimulation increased arterial blood pressure to a plateau 47±19% above baseline levels (Figure 1). During stimulation, cerebral blood flow in the contralateral cortex rose to levels significantly greater than those of the ipsilateral cortex. Despite a tendency for increased flow in the contralateral cortex, changes from baseline CBF did not reach statistical significance on either side of the brain. Both ipsilateral and contralateral resistances increased in response to stimulation, with the increase in ipsilateral resistance being greater than that of the contralateral cortex.

### Autoregulation

To assess the role of the SCG in autoregulation of CBF, preterm and two-week-old lambs were subjected to alterations in arterial blood pressure while CBF was recorded continuously. Blood pressure was changed first with both SCGs intact and then repeated after surgical removal of one of the SCGs.

Preterm lambs had significantly lower baseline mean arterial blood pressures than two-week-old lambs (58±9 mmHg vs. 74±8 mmHg, respectively, p<0.01, t-test). However, within each group baseline mean arterial blood pressures prior to each intervention were comparable before and after removal of the SCG (58±9 mmHg vs. 56±9 for preterm lambs and 74±8 vs. 75±10 for two-week-old lambs). The effect of the blood pressure interventions on upper body arterial blood pressure and CBF are given in Table 2 for preterm lambs and in Table 3 for two-week-old lambs. The administered blood pressure changes before and after SCG removal did not differ significantly, with the exception of the 25% brachiocephalic and aorta occlusions in the two-week-old lambs, when a lower arterial blood pressure was obtained pre- vs. post-SCG removal (See Table 3, p<0.05, t-test). All blood pressure interventions resulted in a significant change in CBF, with the exception of the 25% aorta occlusions in the two-week-old lambs (1-way ANOVA). In preterm lambs, there were no significant differences between changes in CBF on the two sides of the brain, even after removal of one SCG. In two-week-old lambs changes in CBF were also similar on both sides of the brain except following the largest increases in CBF, observed following the norepinephrine infusion, when flow increased significantly more in the side of the brain ipsilateral to the removed SCG.

**Table 2:** Preterm lamb response to changes in blood pressure.

	Prior to Unilateral SCG Removal			After Unilateral SCG Removal		
Intervention	Blood Pressure Change (mmHg)	Ipsilateral CBF Change(% baseline)	Contralateral CBF Change (% baseline)	Blood Pressure Change (mmHg)	Ipsilateral CBF Change (% baseline)	Contralateral CBF Change (% baseline)
100% BC Occlusion	–30±4	–69±8	–67±6	–27±3	–41±15	–51±10
50% BC Occlusion	–17±3	–32±6	–38±7	–14±3	–25±9	–32±10
25% BC Occlusion	–8±1	–14±6	–20±8	–11±2	–16±6	–24±11
100% Aorta Occlusion	18±3	34±9	28±10	25±3	38±8	41±11
50% Aorta Occlusion	15±2	30±6	30±7	16±2	27±6	27±10
25% Aorta Occlusion	9±2	20±6	18±5	10±2	12±7	14±5
NE bolus	28±4	50±12	42±10	30±4	76±19	62±21
AT II bolus	26±3	40±8	35±9	21±3	43±11	33±9

SCG  =  superior cervical ganglion, BC  =  Brachiocephalic artery, NE  =  norepinephrine, AT II  =  angiotensin 2, CBF  =  cerebral blood flow.

All interventions resulted in significant changes from baseline.

All blood pressure and CBF changes after unilateral SCG removal did not differ significantly from pre-SCG removal responses (t-test for blood pressure, 1-way ANOVA for CBF).

**Table 3:** Two-week-old old lamb response to changes in blood pressure.

	Prior to Unilateral SCG Removal			After Unilateral SCG Removal		
Intervention	Blood Pressure Change (mmHg)	Ipsilateral CBF Change (% baseline)	Contralateral CBF Change (% baseline)	Blood Pressure Change (mmHg)	Ipsilateral CBF Change (% baseline)	Contralateral CBF Change (% baseline)
100% BC Occlusion	–48±8	–69±8	–67±7	–40±5	–39±9	–51±10
50% BC Occlusion	–31±7	–33±9	–38±7	–23±4	–39±9	–44±6
25% BC Occlusion	–16±2	–17±12	–17±9	–7±2§	–12±9	–19±12
100% Aorta Occlusion	50±11	14±7	19±8	41±4	36±11	44±14
50% Aorta Occlusion	31±5	18±8	20±11	28±2	47±18	37±21
25% Aorta Occlusion	6±3	7±6	9±7	15±1§	13±11	–1±5
NE bolus	109±13	15±7	18±7	110±6	97±16* †	39±12
AT II bolus	47±10	16±6	22±9	41±7	39±8	44±6

SCG  =  superior cervical ganglion, BC  =  Brachiocephalic artery, NE  =  norepinephrine, AT II  =  angiotensin 2, CBF  =  cerebral blood flow.

* =  significant difference from contralateral response after SCG removal, p<0.01, 1-way ANOVA with Bonferroni post-test.

† =  significant difference from corresponding change before SCG removal, p<0.01, 1-way ANOVA with Bonferroni post-test.

§ =  significant difference from corresponding blood pressure change before SCG removal, p<0.05, t-test.

**Figure 2:**
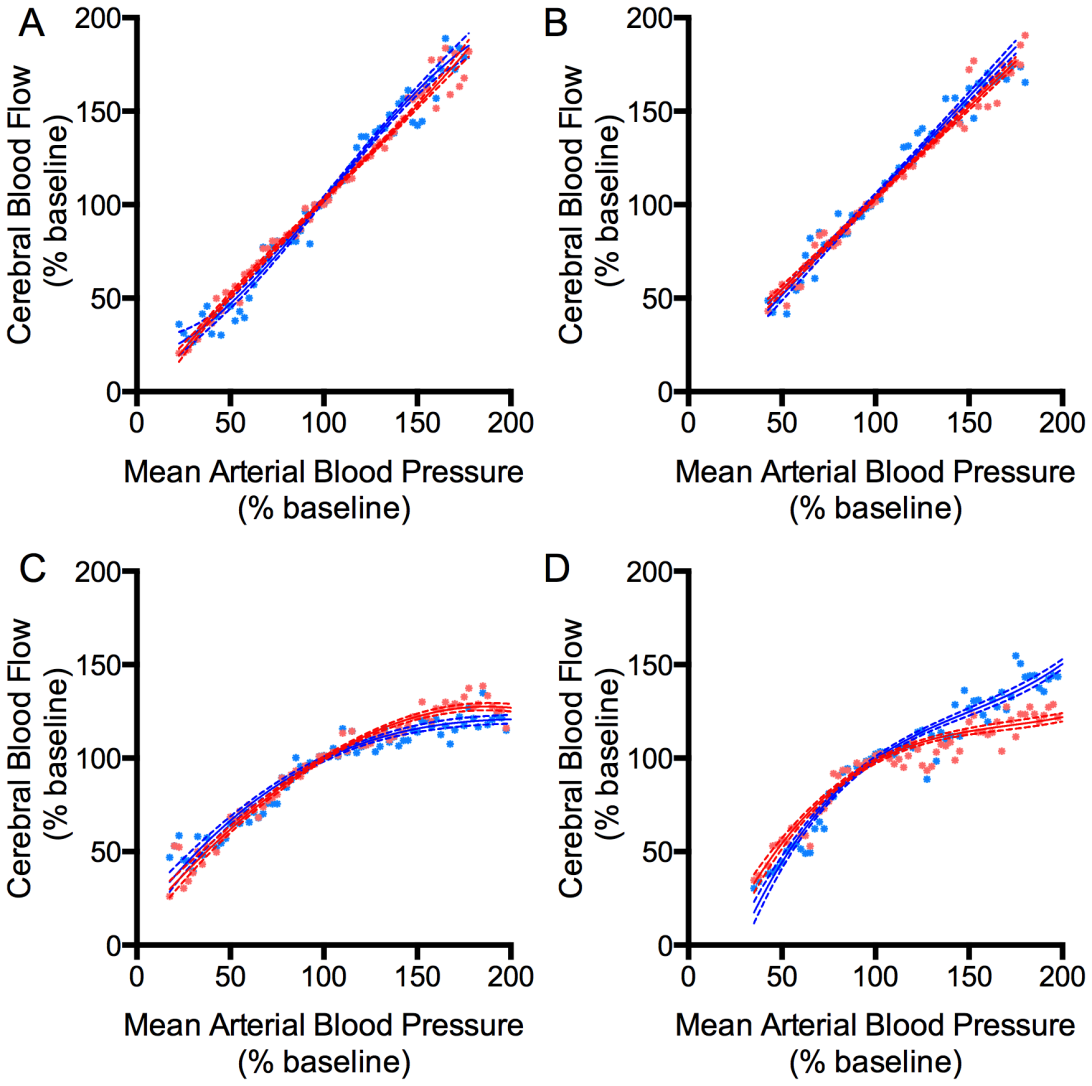
Relationship between mean arterial blood pressure and CBF in preterm (A and B, *n*  =  6) and two-week-old lambs (C and D, *n*  =  6). In preterm lambs, CBF was pressure-passive both before (A) and after (B) removal of one SCG. Cerebral blood flow was similar in both sides of the brain (blue  =  ipsilateral to the side where the SCG was removed, red  =  contralateral) before and after removal of the SCG. Two-week-old lambs demonstrated autoregulation of blood flow in response to hypertension in both sides of the brain prior to SCG removal (C). Following SCG removal (D), autoregulation was attenuated at the upper range of blood pressures on the ipsilateral side of the brain compared to the contralateral side. Lines represent linear (preterm) or third-order polynomial (two-week-old) regressions with ±95% confidence intervals. Data are mean ± SE.*  =  significant difference from contralateral cortex (p<0.01, 2-way ANOVA with Bonferroni post-test).

The relationship between mean arterial blood pressure and CBF across all ranges of arterial blood pressure studied before and after removal of the SCG is shown in Figure 2. In preterm lambs prior to removal of the SCG (Figure 2A), this relationship was linear across the entire range of blood pressures, indicating a lack of cerebral autoregulation in these animals. The same was true following removal of the SCG, and there were no significant differences between the two sides of the brain (Figure 2B). In contrast, prior to SCG removal, increases in CBF in two-week-old lambs were relatively small across the range of elevated mean arterial blood pressures which ranged from ∼50 to as high as 165 mmHg (Figure 2C). Following removal of the SCG, at the highest levels of hypertension CBF increased significantly more on the side of the brain ipsilateral to the removed SCG compared to the contralateral side (Figure 2D), suggesting SCG removal resulted in a unilateral attenuation of cerebral autoregulation. The relationship between arterial blood pressure and CBF was more accurately fit to a linear equation in the preterm lambs, while the two-week-old lamb data demonstrated a better fit to a third-order polynomial, consistent with the absence and presence of cerebral autoregulation in preterm and two-week-old animals, respectively.

## Discussion

The present experiments demonstrate that the sympathetic innervation of the cerebral vasculature serving the cortex is functionally intact and, if stimulated, modulates CBF in both preterm lambs following cesarean delivery and two-week-old lambs born naturally at term. The work also demonstrates a lack of cerebral autoregulation in anesthetized and mechanically ventilated preterm lambs following cesarean delivery, in contrast to similarly instrumented and anesthetized two-week-old lambs in which effective autoregulation was observed. Finally, the experiments indicate that the SCG does contribute to autoregulation in the two-week-old lambs, but that it is only essential during episodes of supraphysiological hypertension.

### Sympathetic stimulation causes cerebral vasoconstriction

The development of functional adrenergic innervation of vasculature occurs in stages, with postsynaptic components of the system maturing before presynaptic components are in place [29]. The contractile response to exogenous norepinephrine is more pronounced in cerebral arteries isolated from preterm and term baboons [30], preterm human infants [31], term fetal sheep [32], and in pial window preparations in newborn piglets [16] and preterm and newborn lambs [17] compared to adults of the same species, suggesting that the postsynaptic function is intact even before birth.

The time course of maturation of presynaptic sympathetic systems is less clear. The contractile response of isolated middle cerebral arteries to transmural electrical stimulation increases with developmental age from near term fetal sheep to newborns and adults [33]. Sympathetic stimulation *in vivo* results in decreased CBF in newborn lambs [15] and piglets [34] suggesting the presynaptic apparatus is functional, although *in vivo* comparisons of presynaptic function in newborns and adults of the same species have not been performed. Although we did not observe a significant decrease in CBF in the two-week-old lambs, our findings are consistent with these previous newborn reports in that we also observed a significant increase in ipsilateral cerebrovascular resistance to flow in response to unilateral SCG stimulation (Fig. 1). Wagerle *et al*, were the first to study the effects of sympathetic stimulation on the cerebral vasculature in preterm fetal animals. In their study using acutely instrumented fetal sheep with the umbilical circulation still intact, pial arteriole diameter decreased during electrical stimulation of the SCG [17], although the effect on CBF was uncertain since pial arteriole constriction might have been compensated for by dilation of downstream resistance vessels resulting in no appreciable change in overall flow. The present study extends this previous work by demonstrating the capacity of sympathetic nerves to increase cerebrovascular resistance and decrease CBF in the intact preterm lamb.

We found that the effects of unilateral stimulation of the SCG on CBF were limited to the ipsilateral cortex, with no significant crossover effect to the contralateral hemisphere. In contrast, there is evidence of crossover in adult cats and rabbits where bilateral denervation has a significantly greater effect on basal CBF than unilateral denervation. Likewise, bilateral sympathetic stimulation results in a greater increase in cerebrovascular resistance in either cortex compared to unilateral stimulation [35]. The reason for the lack of crossover effect in our studies could be related to a lack of myelination. Lambs begin to deposit myelin at 80 days gestation followed by two periods of accelerated myelination, occurring approximately 20 days prior to birth and then again 20 days after birth [36], well after the age of either group of lambs studied in these experiments.

### Lack of autoregulation in preterm lambs

The absence of autoregulation in the preterm lambs in our study was unexpected. Previous reports have demonstrated autoregulation to be intact in chronically instrumented fetal sheep as early as 92 [37], 110 [28], 118 [1], and 125 days [38] of gestation. An obvious difference between these studies and ours is the fact that our lambs were delivered by C-section, anesthetized, and mechanically ventilated whereas all previous work in preterm sheep has been performed using fetuses instrumented *in utero*. These procedures, which are not uncommon in the clinical care of infants, are likely to have resulted in marked changes in the concentrations of vasoactive compounds such as prostaglandins, adenosine, catecholamines, and nitric oxide. In addition, arterial and brain tissue oxygen tensions rise dramatically during the birth transition, which likely has potent effects on concentrations of a variety of vasoactive metabolites produced within the brain. The overall effect of parturition on autoregulation is largely unstudied, yet of clear clinical importance.

Another key difference between our study and previous reports in preterm lambs is the use of anesthetic and paralytic agents, as well as mechanical ventilation. However, ketamine [39], vecuronium [40], and mechanical ventilation have been found to not disturb autoregulation in other studies, as well as in the similarly instrumented two-week-old lambs in the present study. Muller *et al* were able to detect autoregulation in chronically instrumented fetal lambs younger than in the present study using a similar laser Doppler instrumentation [28], raising the possibility that autoregulation is temporarily lost due to some other factor affected by delivery such as the rapid change in tissue PO_2_, or changes in circulating concentrations of a number of endogenous vasoactive compounds. It is also possible that insertion of the laser Doppler probes in our preterm lambs caused injury that disrupted local autoregulation during the course of the study, although this did not occur in the two-week-old lambs with similar surgical instrumentation. Importantly, results in our animal model are consistent with clinical studies of preterm infants that suggest that autoregulation is markedly impaired in the first few hours and days following birth [41].

### Effect of SCG removal on autoregulation

The preterm lambs in our study lacked measurable autoregulation. Therefore, we were unable to examine the effects of SCG removal on autoregulation in this group. In the two-week-old lambs, however, autoregulation effectively attenuated increases in CBF even during acute hypertension (Figure 2C). Our finding that removal of the SCG resulted in a more pressure-passive increase in ipsilateral CBF during hypertension indicates that the SCG does contribute to autoregulation of flow during brief hypertensive episodes. This result is consistent with previous studies in newborn piglets [8] and adult cats [9], monkeys [7], and baboons [42], in which unilateral removal of the SCG resulted in impaired autoregulation. The loss of autoregulation is especially evident under conditions of hypertension in these animal models [9], [42]. Notably, the loss of autoregulation in two-week-lambs in the present studies was only significant at extremely elevated arterial blood pressures, suggesting that although the SCG may extend the range of autoregulation to greatly elevated arterial blood pressures, it is not essential for autoregulation within the physiological range of blood pressures. Whether the SCG does contribute to autoregulation within the physiological range but its loss was compensated by other mechanisms cannot be determined from the current study.

### Study Limitations

Laser Doppler flowmetry is limited in that it can only detect changes in flow in a relatively small volume of tissue (∼2 mm^3^). In this study the probe was positioned in the parietal cerebral cortex, approximately 0.5 cm below the surface of the brain, a location in the forebrain where blood flow has been shown to be under the influence of sympathetic input [43]. However, caution is necessary in extrapolating our findings to other parts of the brain since the magnitude of both sympathetic control of CBF and autoregulation are heterogeneous across the various regions of the brain. In addition, although our use of norepinephrine and angiotensin II enabled the study of higher arterial blood pressures than could be obtained by mechanical methods, we cannot exclude the possibility that these agents may have altered autoregulation by direct effects on the cerebral vasculature.

Both the SCG stimulations and interventions in arterial blood pressure in our study were of relatively short duration in order to ensure that the lambs remained in stable condition throughout the experiment. However, this limited time prevented assessment of long-term decreases in CBF, which may be limited by a time-dependent ‘escape’ mechanism in some animal models [43]. Although the interventions were brief, it is still possible that interventions early in the experiment may have altered responses to subsequent ones, although this effect would not necessarily invalidate the comparison of ipsilateral and contralateral responses. It is also possible that the administration of 100% inspired O_2_ to the lambs in both groups may have attenuated cerebral autoregulation. However, this possibility appears unlikely as studies of the administration of high inspired O_2_ in humans find no change [44] or even improvements [45] in cerebral autoregulation, with no studies reporting decreases in autoregulation in response to high inspired O_2_. Other limitations include failure to monitor and maintain glucose and renin levels, and an uneven ratio of right-to-left SCG chosen for stimulation and removal, which may have biased the results if the effects of the two ganglia are asymmetrical. Also, the experimental preparation required instrumentation that may have injured cerebral blood vessels, altering blood flow responses. And finally the sheep is a species whose young are quite mature at birth and vascular control is likely to develop more slowly in infants. Furthermore, the anatomy of the vasculature supplying the sheep brain differs from that of the human as blood passes through a carotid rete before reaching the Circle of Willis in the sheep. The effect of these species differences on autoregulation and the role of the SCG are not known.

### Clinical Perspectives

Dysregulation of CBF, resulting in intraventricular hemorrhage and periventricular leukomalacia, is a common problem in the care of premature infants. The present study demonstrates that, although cerebral autoregulation is intact in the fetal sheep *in utero* at an early stage of development, it may be lost with premature delivery and following treatments common to the clinical care of premature infants, such as intubation and mechanical ventilation. The present findings indicate that sympathetic control of cerebral vascular resistance during marked hypertension may be limited immediately following preterm birth. Furthermore, excessive sympathetic activation may potently decrease CBF after birth, contributing to inadequate flow observed in some pathologies.
